# One-Year Prospective Study on Smartphone-Based Coefficient of Variation Analysis of Seated Stepping Movements for Fall Risk Prediction in Older Adults

**DOI:** 10.3390/s26010080

**Published:** 2025-12-22

**Authors:** Daisuke Sudo, Naoki Wada, Naoko Matanoki, Yuko Mine, Yoshiyuki Kobayashi

**Affiliations:** 1 National Institute of Advanced Industrial Science and Technology (AIST), c/o Kashiwa II Campus, University of Tokyo, 6-2-3 Kashiwanoha, Kashiwa 277-0882, Japan; 2 SOMPO Care Inc., Shinagawa Seaside East Tower, 4-12-8 Higashi-Shinagawa, Shinagawa-ku, Tokyo 140-0002, Japan; 3 University of Occupational and Environmental Health, 1-1 Iseigaoka, Yahatanishi-ku, Kitakyushu-shi 807-8555, Japan

**Keywords:** coefficient of variation, fall risk, seated stepping movements, smartphone

## Abstract

Older adults with a recent fall history tend to have larger variability in stepping than those without a fall history. In this study, we examined whether variability in step timing—defined as the coefficient of variation (CV) of step timings during seated stepping exercises—can identify individuals at higher risk of falling. The CV was measured at baseline (initial assessment), and fall occurrences were tracked over one year among 58 older adults in 11 senior housing facilities participating in online exercise programs. Participants who experienced falls exhibited marginally higher CV values at baseline than those who did not fall, and those who fell specifically while walking showed significantly higher CVs compared to non-fallers (*p* = 0.035). Logistic regression analysis indicated that the CV significantly predicted walking-related falls (odds ratio = 1.24, *p* = 0.032), and receiver operating characteristic curve analysis yielded an area under the curve of 0.747, suggesting moderate discriminative ability. Including prior fall history in the model further improved predictive performance (AUC = 0.807 for overall falls and 0.925 for walking-related falls), suggesting that combining CV with prior fall history enhances predictive performance. These findings suggest that evaluating timing variability during seated stepping exercises, especially when combined with prior fall history, may be a useful indicator for predicting fall risk over the following year without exposing individuals to fall hazards during assessment.

## 1. Introduction

Falls increase the physical and economic burden on older adults and reduce their quality of life, making early intervention essential. Recently, the incidence of falls among older adults has increased, with over 684,000 fall-related deaths and nearly 37 million medical interventions occurring annually worldwide [1]. Falls can have severe consequences, including femoral fractures, hospitalization, and even death [2,3,4]. In the United States, the economic burden of falls on older adults has increased. In 2018, approximately 90% of the 2.4 million emergency department visits among adults aged 65 years and older were caused by unintentional falls [5,6]. Therefore, falls are associated with decreased quality of life and increased healthcare costs, making appropriate preventive measures essential [7,8,9,10]. In addition, falls deteriorate health, leading to increased hospitalization and medical visits [11,12]. Considering these effects, early and effective fall risk assessments are required.

Various methods for assessing fall risk, such as the timed up-and-go (TUG) test and gait speed, are widely used in clinical settings [13,14]. However, these methods require individuals with diminished physical or cognitive function to perform movements that pose a risk of falling, and they require substantial time and resources from hospital and facility staff, which are often limited [15]. Human gait exhibits variability with each step. Individuals with a higher risk of falling tend to show greater gait variability than those with a lower risk of falling. Neuromuscular dysfunction and impaired sensory feedback mechanisms contribute to increased gait variability in individuals at a high risk of falling [16]. These factors may also influence the variability of other repetitive movements beyond gait [16]. Several studies have analyzed the relationship between gait variability and fall risk, as well as the association between walking speed and stride variability, indicating that variability in walking movements is closely related to physical performance [16,17,18]. However, asking older adults at high risk of falling to walk under various conditions raises safety concerns. Previous research has examined seated stepping exercises in relation to sarcopenia risk, but no prospective studies have investigated the association between variability in seated stepping and fall risk [19]. In addition, studies have explored step tests performed at stipulated tempos, showing that timing errors at slower tempos were significantly correlated with walking speed, suggesting their utility in dynamic balance assessment [20]. Furthermore, results of the Step Test Evaluation of Performance on Stairs (STEPS) were significantly correlated with functional measures such as the Timed Up and Go (TUG) and chair rise tests, indicating its effectiveness in assessing stair negotiation ability and agility [21]. These findings highlight the potential of seated or step-based assessments as safe and practical alternatives for evaluating mobility and fall risk in older adults in clinical practice. However, no prior work has examined whether timing variability during seated stepping can predict future falls, which represents the novelty of our approach. Consequently, the first limitation can be addressed if the fall risk could be assessed through the variability of repetitive movements other than walking performed in a safe environment. Moreover, many senior care facilities have staff shortages, and the workload for each staff member is extremely high [22,23,24]. Consequently, despite their accuracy, conducting tests such as the TUG daily is impractical.

Retrospective studies in both laboratory and field environments have been performed to examine the relationship between the variability of the timings during seated stepping exercises and fall experience risk over the past year [25]. In both settings, older adults with a history of falls in the past year (high fall risk group) exhibited significantly higher coefficient of variation (CV) values than those without a fall history (low fall risk group) [25]. However, because previous studies were retrospective, the association between the variability of timings during seated stepping exercises and fall experience over 1 year after the experiment remains unclear.

In this study, we aimed to clarify the relationship between the variability of the timings during seated stepping exercises performed in an online exercise program at a senior care facility and fall occurrences over the following year. Gait variability is associated with fall occurrence in the following year [26,27,28]; therefore, we hypothesized that individuals with a higher fall risk would also exhibit greater variability in seated stepping motion than those with a lower fall risk.

## 2. Materials and Methods

### 2.1. Participants

We collaborated with SOMPO Care Inc., located in Tokyo, Japan, which operates senior care facilities throughout Japan, and recruited 94 residents from 11 facilities in who regularly participated in online exercise classes. The inclusion criteria were as follows: (1) care level, as defined by the Japanese Ministry of Health, Labour and Welfare, was Care Level 2 or lower (i.e., Care Level 1, 2, Support Level 1, 2, or fully independent) [29], (2) those who regularly participated in the exercise class within the facility, and (3) those who were able to walk without assistive devices or orthoses and reported no pain at the time of measurement. Regular participants were defined as those who attended nearly all sessions of the exercise class as of the baseline assessment. The exclusion criteria were those with uncorrectable visual impairment and a history of trauma or fractures within the past year. A total of 67 residents participated in the baseline survey; however, at the 1-year follow-up survey, nine individuals were excluded because of hospitalization, facility relocation, refusal to participate in the study, or non-participation in the exercise program. Consequently, 58 participants completed the one-year follow-up survey, during which their fall history was assessed based on both self-reports and facility records. According to available records and interviews, none of the hospitalizations were related to falls. All participants received both written and verbal explanations before the experiment and provided informed consent before participation. The study protocol was approved by the AIST Bioethics Committee (approval no. 71160030-E-20230731-008).

**Figure 1:**
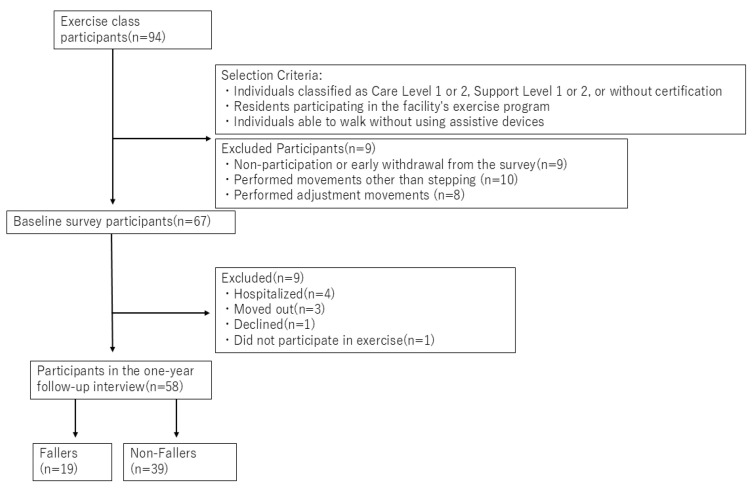
Study flowchart of participant selection and follow-up.

The definition of falls was based on previous research and was defined as “Falling to the same level or a lower surface due to inadvertence, without external force exerted by another person, loss of consciousness, sudden paralysis caused by a stroke, or seizures caused by epilepsy” [30]. Based on the follow-up results, 19 participants were classified as having a fall history, whereas 39 participants had no fall history. A flowchart of the participant recruitment process is shown in Figure 1. Details of follow-up survey, including the hearing and report-based assessment, are described in Section 2.5.

### 2.2. Structure of Online Exercise Class

The online exercise class provided at the care facilities consisted of a 60 min program that included one hydration break. The first 30 min consisted of stretching and training (either with equipment or standing exercises). The next 30 min consisted of aerobic exercises, including seated stepping exercises. These exercises were performed approximately once per second for 16 s. The overall duration and intensity of the program were approximately the same each time.

A large monitor was placed in front of the participants in each care facility, and they were instructed to mimic the trainer’s exercise movements, which were displayed on the screen. During the exercise class, the trainer’s verbal instructions and calls, as well as background music at 120 bpm, were played over the speakers.

### 2.3. Recording of Seated Stepping Motion

**Figure 2:**
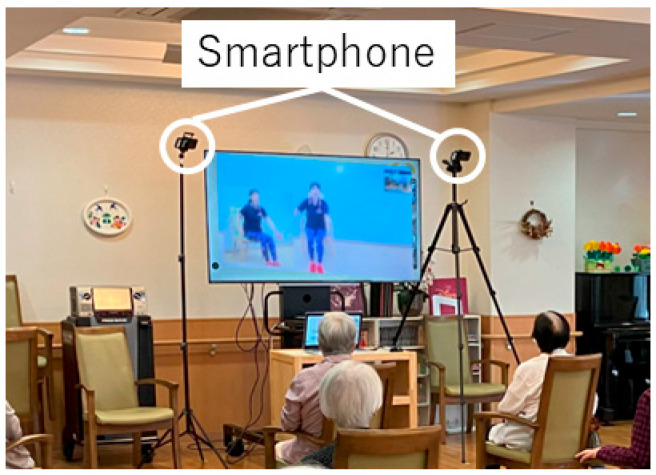
Filming environment. A large monitor displayed exercise videos to the participants, while multiple smartphones were positioned at different angles to capture their movements.

One to three iPhone 14 Pros (Apple Inc.) placed on tripods were used to record the seated stepping motion of participants during the exercise. The devices were mounted on tripods with a height ranging from 160 to 200 cm and positioned 2 to 4 m in front of the participants. Each camera was adjusted to capture the entire body, ensuring visibility of the legs during seated stepping. Depending on the number of participants, one device recorded up to seven participants simultaneously, with a maximum of three devices recording up to 13 participants per session. As the number of participants and the size of the rooms varied between facilities, the number of cameras, camera height, angle, and location were adjusted to record whole-body motion without occlusion. A single camera captured images of up to seven participants. The frame rate was set at 240 Hz. The exercise was filmed from before it began to after it ended to avoid disturbing the class progression, and the part of the seated stepping exercise was extracted and analyzed. An example of the filming setup for an exercise class held at a care facility is shown in Figure 2.

### 2.4. Computation of the Variability of the Timings During Seated Stepping Exercise

**Figure 3:**
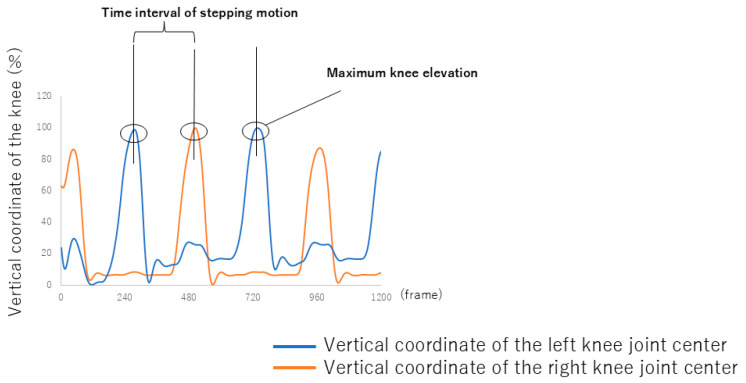
Time-series data of the vertical coordinates of knee joint centers during seated stepping exercises: left (blue) and right (orange) knee joint centers. The peaks in the graph represent the maximum knee elevation for each step motion.

To compute timing variability, we extracted a 16 s segment from the exercise video and removed approximately 3 s from both the beginning and end to avoid transition effects. This resulted in a 10 s clip containing ten consecutive steps from the middle of the session. We identified the lifting point for each step, defined as the moment when the knee reached its maximum vertical height (Figure 3), based on the finalized knee landmark coordinates. For each leg, ten stepping intervals were calculated as the time between two consecutive lifting points of the same leg. The coefficient of variation (*CV*) of these intervals was computed using the following equation:

(1) $$CV=\frac{\sigma }{\mu }\times 100$$

where *σ* is the sample standard deviation of the time intervals, and *μ* is the mean time interval. Knee landmarks were automatically detected using AI-based marker-less motion capture software (Pose-Cap V1.33, Four Assist Co., Ltd., Tokyo, Japan), and manually corrected when necessary using the G-Dig software V1.17 (Four Assist Co., Ltd., Tokyo, Japan).

### 2.5. Fall History Assessment via Interview and Facility Records

Participants were asked whether they had experienced a fall during the one year from the date of their previous visit to the date of the hearing survey. If a participant reported experiencing fall(s), the following additional details were collected: number and timing of falls; location (e.g., private room, around the bed, toilet, hallway, entrance, dining area, outdoors, sidewalk, or other); activity at the time of the fall (e.g., walking, walking without a cane, standing up, opening a door, reaching for an object at a high place, or other); direction of the fall (e.g., forward, backward, left, right, diagonally forward, diagonally backward, or other); pain or injury status (e.g., none, minor injury, hospitalization, or other); and changes or additions to walking aids (e.g., none, cane, walker, rollator, handrail, or other) In addition to self-reports, fall history was also confirmed through facility records to enhance data reliability. In cases where discrepancies were found between self-reported fall history and facility records, participants were classified as having a fall history if either source indicated a fall.

### 2.6. Statistical Analysis

Statistical analyses were performed using IBM SPSS Statistics (v. 29, IBM Corp., Armonk, NY, USA) for comparison and CV of stepping timings between the groups. First, the Shapiro–Wilk test was used to assess data normality. Levene’s test was used to evaluate the homogeneity of variances before applying the *t*-test. When equal variances were confirmed (*p* > 0.05), the standard independent sample *t*-test was used; otherwise, Welch’s *t*-test was applied. If the data followed a normal distribution, an independent sample *t*-test was conducted to compare fallers and non-fallers. The significance level was set at 5% (*p* < 0.05), with 10% (*p* < 0.10) considered marginally significant [31]. Cohen’s d was calculated to determine the effect size of the differences between groups. According to the guidelines, an effect size of 0.2 is considered small, 0.5 is medium, and 0.8 or higher is large [32].

Additionally, binary logistic regression analyses were conducted to evaluate the predictive ability of the timing variability (CV) for fall occurrence. Age was included as a covariate. Odds ratios (OR) with 95% confidence intervals (CIs) are reported. Receiver operating characteristic (ROC) curves were generated, and the area under the curve (AUC) was calculated to assess discriminative performance.

## 3. Results

Patient characteristics are presented in Table 1. Table 2 presents the circumstances under which falls occurred in the faller group (n = 19), with a total of 23 reported fall events. The most common activity during which falls occurred was walking indoors (9 events, 39.13%), followed by outdoor walking (4 events, 17.38%). Other fall situations included bus boarding, standing up with support, falling from bed, dressing, opening a door, and reaching for an object in the cafeteria—each accounting for one event (4.35%). There was one unwitnessed fall where the participant was found on the floor, and three events (13.04%) had unknown details.

**Figure 4:**
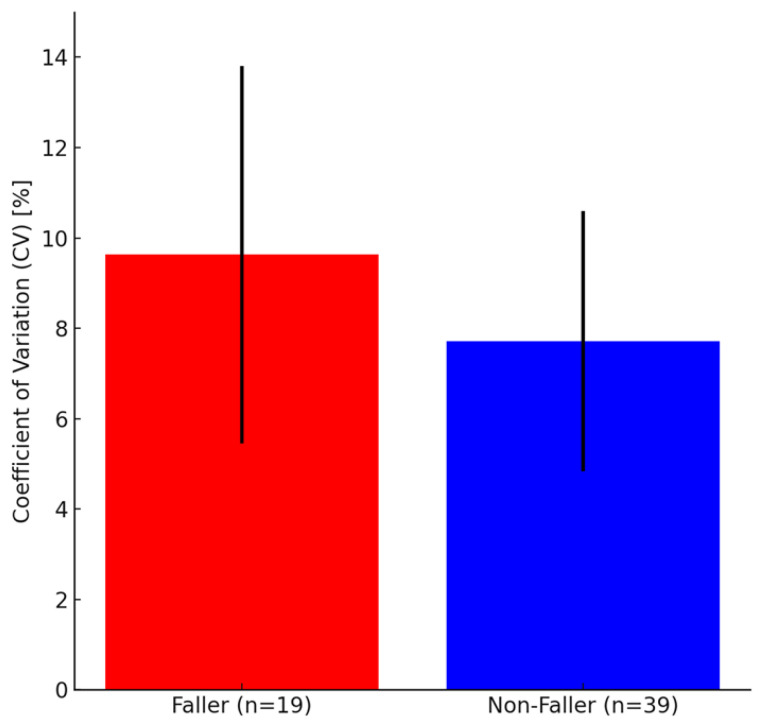
Coefficient of variation (CV) values in participants with and without fall history after one year. The fallers (red bar) had a higher CV value than the non-fallers (blue bar). Although the difference was not significant (*p* = 0.083), the effect size (d = 0.57) suggests a moderate effect. Error bars represent standard deviations. Coefficient of variation (CV) values for all fallers versus non-fallers after 1 year. Fallers include participants who experienced at least one fall, regardless of context or activity.

**Figure 5:**
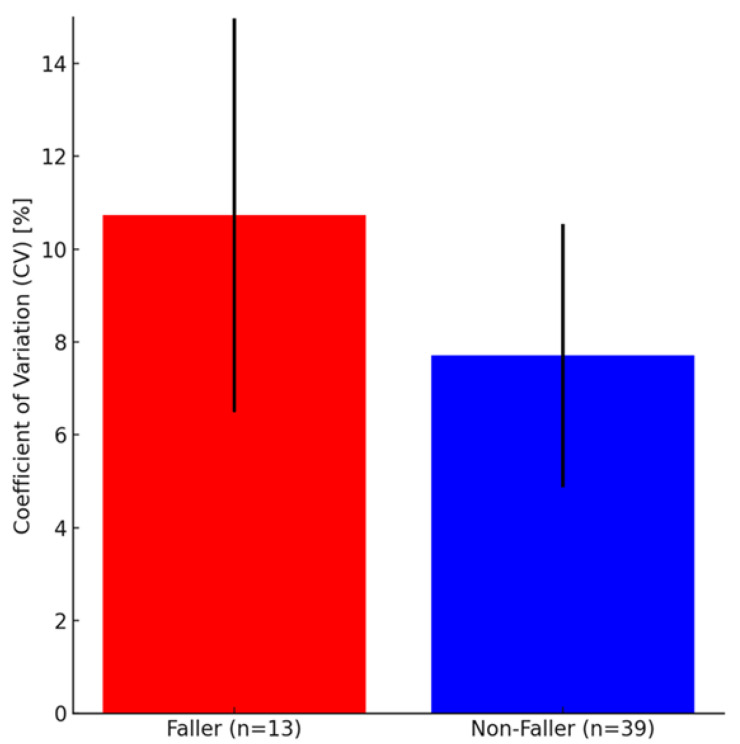
Coefficient of variation (CV) values in participants with and without a fall history after one year. The fallers (red bar) had a higher CV value than the non-fallers (blue bar). The difference was significant (*p* < 0.05), indicating that individuals with a history of falls exhibited greater gait variability. The effect size (d = 0.94) suggests a large effect. Error bars represent standard deviations. Coefficient of variation (CV) values for walking fallers versus non-fallers after one year. Fallers include only participants whose falls occurred specifically while walking.

Comparison of CV values measured at baseline one year prior between the faller and non-faller groups showed a marginally significant difference with a large effect size (*p* = 0.083, d = 0.57) (Table 3, Figure 4). Significant differences were observed between non-fallers and fallers when fallers were limited to only those 13 who fell while walking (i.e., excluding those who fell while not walking) (*p* = 0.035, d = 0.94) (Table 4, Figure 5).

**Figure 6:**
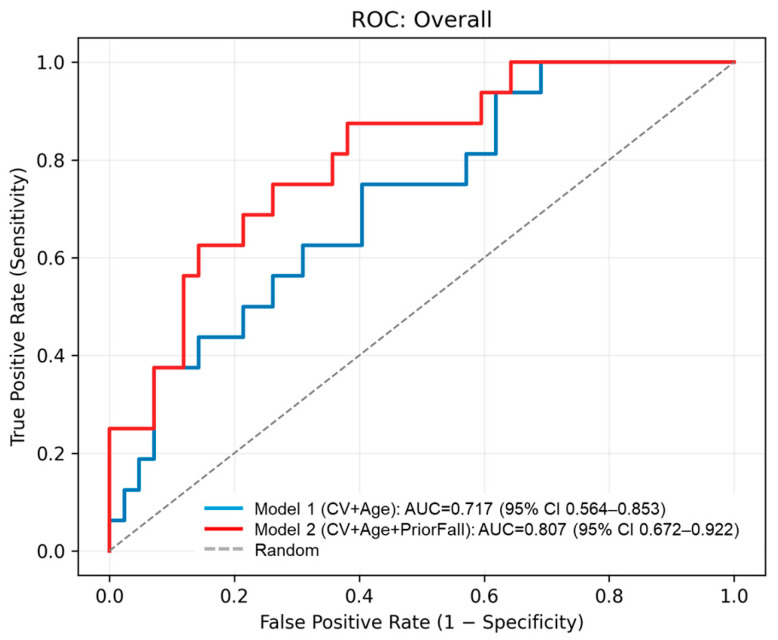
Receiver operating characteristic (ROC) curves for predicting overall fall occurrence based on two logistic regression models. The blue line represents Model 1 (CV + Age), with an AUC of 0.717 (95% CI: 0.564–0.853). The red line represents Model 2 (CV + Age + Prior Fall History), showing improved performance with an AUC of 0.807 (95% CI: 0.672–0.922). The dashed line indicates random classification.

**Figure 7:**
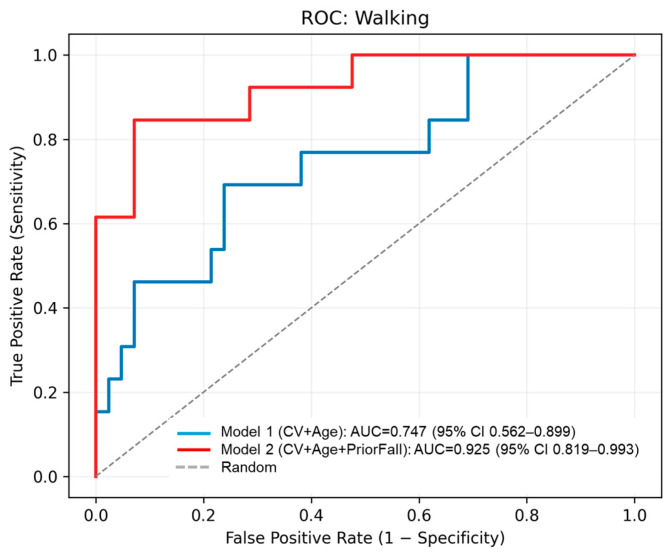
Receiver operating characteristic (ROC) curves for predicting walking-related falls. The blue line represents Model 1 (CV + Age), with an AUC of 0.747 (95% CI: 0.562–0.899). The red line represents Model 2 (CV + Age + Prior Fall History), showing improved performance with an AUC of 0.925 (95% CI: 0.819–0.993). The dashed line indicates random classification.

To further evaluate the predictive performance of the CV and the effect of prior fall history, we conducted binary logistic regression analyses with fall occurrence as the dependent variable. Model 1 included CV and age as predictors, while Model 2 additionally included prior fall history. CV tended to be associated with overall fall occurrence (OR = 1.17, 95% CI: [0.98, 1.40], *p* = 0.080), and age showed a similar trend (OR = 1.13, 95% CI: [0.98, 1.31], *p* = 0.088). Prior fall history was a significant predictor in Model 2 (OR = 6.39, 95% CI: [1.46, 27.94], *p* = 0.028). The CV remained a significant predictor when restricting falls to those occurring during walking (OR = 1.24, 95% CI: [1.02, 1.52], *p* = 0.032). ROC analysis yielded an AUC of 0.717 for overall falls and 0.747 for walking-related falls in Model 1, and the AUC improved to 0.801 when prior fall history was included (Figure 6 and Figure 7). Table 5 summarizes these logistic regression results. These findings provide preliminary evidence that supports the potential of the CV as a predictor of future falls beyond prior fall history. However, the small sample size limits generalizability.

## 4. Discussion

This study shows that the CV of the timing of seated stepping exercises was notably higher in older adults who experienced a fall within 1 year after baseline assessment, with marginal statistical significance. Logistic regression analysis further indicated that the CV was a significant predictor of walking-related falls (OR = 1.24, *p* = 0.032), and ROC analysis yielded an AUC of 0.747, suggesting moderate discriminative ability. Furthermore, when prior fall history was included in the logistic regression model (Model 2), predictive performance improved substantially (AUC = 0.807 for overall falls and 0.925 for walking-related falls), suggesting that combining CV with prior fall history enhances predictive performance. Combining CV with prior fall history may offer a more robust approach for fall risk screening in clinical and care settings.

However, the evidence remains preliminary and should be interpreted with caution given the small sample size and limited adjustment for potential confounders such as neuromuscular or sensory factors. Moreover, when focusing specifically on participants who experienced falls while walking, the CV was significantly higher compared to that of non-fallers, with a large effect size, suggesting that seated stepping variability may be particularly relevant for identifying individuals at risk of walking-related falls. The finding that greater variability in the timing of the seated stepping exercise was observed in fallers is consistent with those of previous studies reporting that gait variability is higher in individuals with a history of falls [27,28]. Several studies have further demonstrated that variability-based metrics, such as stride-to-stride variability and variability in step width, are significant predictors of future falls [17,18]. The results of this study suggest that multifaceted factors may influence the variability of timings during seated stepping exercises. Although we did not collect individual participant histories related to neuromuscular, sensory, or psychological conditions, such factors have been suggested to contribute to movement variability and fall risk [16].

Fall risk assessment based on the variability of timings during seated stepping exercises may solve the two above-mentioned limitations of existing fall risk assessment methods, namely, the risk of inducing falls when assessing high-risk individuals and the lack of time and personnel available in care settings. Most falls occur during walking [33]; therefore, in conventional assessments, older adults must be exposed to fall risk to evaluate their fall risk. In addition, falls in care facilities can lead to legal disputes and litigation, further emphasizing the need for safer fall risk assessment methods. In the United States, many older adults cannot access necessary caregiving services, with 94% of local agencies reporting staffing shortages [22]. In rural areas, securing a caregiving workforce is particularly challenging compared to urban regions, and this shortage substantially affects the quality-of-care services [23]. Therefore, assessing fall risk based on the movement characteristics observed during routine seated exercises can address these challenges, as indicated in this study.

Variability in knee height could further enhance the accuracy of fall risk assessment; however, this study focused solely on analyzing variability in the timing of seated stepping exercises. This decision was based on two key considerations. First, camera calibration is required before use if knee height is included as an evaluation metric. However, if only timing is evaluated as a parameter, calibration can be omitted as long as the knee is visible. As care facilities do not always have staff familiar with handling technical devices, a simple and easy-to-use approach for practical implementation was prioritized. In general, motion variability can be assessed using multiple parameters, including knee height and step distance, joint angles, or center-of-gravity sway. However, we deliberately focused on temporal variability (i.e., coefficient of variation in timing) to prioritize feasibility in care settings. Second, if the timing is the only evaluated parameter, alternative sensor systems such as inertial measurement units or strain gauges could also be an option. By focusing solely on timing, easier adaptability to non-camera-based sensor systems is ensured, making the method more widely applicable. However, some facilities, residents, or family members may be concerned about privacy and may be reluctant to use cameras.

The variability in timing during seated stepping exercises may indicate risks beyond falls. For example, cognitive decline is associated with increased variability in reaction time [34], and Parkinson’s disease is characterized by increased gait variability [35,36]. While these associations might be leveraged in clinical practice, their application to seated stepping remains hypothetical and requires further validation. Future studies are needed to determine whether the variability in timing during seated stepping can serve as an early indicator for conditions such as Parkinson’s disease and dementia.

This study had several limitations. First, the proposed method alone cannot specify the types of falls that are of particular concern. Yamada [37] developed a method that determines whether muscle strength, balance ability, or cognitive function are the primary factors contributing to fall risk based on TUG results. In the future, as more data are accumulated using the proposed method, key factors that require attention based on the observed trends can be identified. However, a practical approach is to use this method as an initial screening tool and integrate it into more detailed assessments. Second, fall history was collected through a hearing survey conducted one year later, which relied on participants’ memories. Consequently, recall bias may have occurred, leading to missed falls or incorrect reports [38]. However, because the participants were facility residents, available facility records and follow-up by staff could complement participants’ reports, likely improving the accuracy compared to previous studies conducted with community-dwelling older adults. Nonetheless, the possibility of reporting errors cannot be excluded. Third, timing variability was assessed using a single baseline session, which did not allow to account for session-to-session variability. This could have affected the stability of CV estimates. Future studies should incorporate multiple baseline assessments to capture intra-individual variability and improve predictive robustness. Fourth, knee landmarks that artificial intelligence (AI) misdetected during the data analysis were manually corrected, which cannot be performed by caregivers in long-term care facilities. However, with recent advancements in AI technology and skeletal detection techniques, the implementation of more accurate and automated analysis models may minimize the need for these types of data correction in the near future. Finally, the relatively small sample size (58 participants completed follow-up, with subgroup analyses involving only 13 fallers) may limit statistical power and the generalizability of the findings. Future studies with larger cohorts are needed to validate our results.

## 5. Conclusions

This prospective study clarified the relationship between the variability of timings during a seated stepping exercise performed in an online exercise program at a senior care facility and fall occurrences over the following year. Older adults with larger variability (i.e., larger CV) in the seated stepping exercise had a higher fall risk after one year. The results of this study could lead to the development of new, safe, and practical methods for fall risk assessment within routine exercise programs conducted in senior care facilities. Collaboration with long-term care facilities will continue to improve the current system, making it more convenient to use and enhancing data collection processes to improve the accuracy of the risk assessment model.

## Figures and Tables

**Table 1:** Participant characteristics.

	Fallers n = 19	Non-Fallers n = 39
Age	89.68 ± 3.28	84.52 ± 6.21
Sex (male/female)	4/15	5/34
Height (cm)	151.12 ± 12	152.35 ± 6.35
Weight (kg)	48.48 ± 8.11	51.86 ± 8.03
Body mass index (kg/m^2^)	21.12 ± 2.41	22.33 ± 3.05

**Table 2:** Circumstances of falls in the faller group (23 events reported by 19 fallers).

Fall Circumstance	23 Fall Events	%
Walking (indoor)	9	39.13
Walking (outdoor)	4	17.38
Bus boarding	1	4.35
Standing up with support	1	4.35
Falling from bed	1	4.35
While putting on pants	1	4.35
Opening a door	1	4.35
Reaching for an object in cafeteria	1	4.35
Unwitnessed fall, found on the floor in the room	1	4.35
Unknown details	3	13.04

**Table 3:** Comparison of coefficient of variation (CV) between fallers and non-fallers.

Variable	Fallers (n = 19)	Non-Fallers (n = 39)	*p*-Value	95% Confidence Interval (Lower, Upper)	Effect Size (d)
CV	9.62 ± 4.17	7.71 ± 2.87	0.083	(−0.26, 4.09)	0.57

Comparison of the CV between non-fallers and all fallers in the study population. All fallers include participants who experienced at least one fall, regardless of the context or activity at the time of the fall.

**Table 4:** Comparison of coefficient of variation (CV) between walking fallers and non-fallers.

Variable	Walking Fallers (n = 13)	Walking Non-Fallers (n = 39)	*p*-Value	95% Confidence Interval (Lower, Upper)	Effect Size (d)
CV	10.73 ± 4.23	7.71 ± 2.84	0.035	(0.23, 5.79)	0.94

Comparison of the CV between non-fallers and fallers who fell during walking. Fallers include only participants whose falls occurred specifically while walking.

**Table 5:** Logistic regression results for fall occurrence (Model 1 vs. Model 2).

Condition	Variable	Model 1 OR (95% CI)	*p*-Value	Model 2 OR (95% CI)	*p*-Value
Overall Falls	CV	1.172 (0.981–1.404)	0.080	1.110 (0.910–1.354)	0.301
	Age	1.132 (0.982–1.305)	0.088	1.156 (0.992–1.346)	0.063
	PriorFall	—	—	6.395 (1.460–27.947)	0.028
Walking Falls	CV	1.244 (1.018–1.521)	0.032	1.078 (0.796–1.461)	0.629
	Age	1.115 (0.952–1.307)	0.177	1.231 (0.974–1.555)	0.081
	PriorFall	—	—	unstable	0.050

Notes: Model 1 = CV + Age; Model 2 = CV + Age + PriorFall. For walking falls, PriorFall showed quasi-complete separation; OR not interpretable. Model fit: Nagelkerke R^2^ = 0.322 (Overall, Model 1), 0.674 (Walking, Model 2); Hosmer–Lemeshow *p*-values: 0.549 (Overall), 0.952 (Walking).

## Data Availability

The data and algorithms presented in this study are available from the corresponding author upon reasonable request.

## References

[1] World Health Organization (WHO) (2021). Falls. https://www.who.int/news-room/fact-sheets/detail/falls.

[2] Kannus P., Parkkari J., Koskinen S., Niemi S., Palvanen M., Järvinen M., Vuori I. (1999). Fall-induced injuries and deaths among older adults. JAMA.

[3] Hill K., Schwarz J., Flicker L., Carroll S. (1999). Falls among healthy, community-dwelling, older women: A prospective study of frequency, circumstances, consequences and prediction accuracy. Aust. N. Z. J. Public Health.

[4] Parkkari J., Kannus P., Palvanen M., Natri A., Vainio J., Aho H., Vuori I., Järvinen M. (1999). Majority of hip fractures occur as a result of a fall and impact on the greater trochanter of the femur: A prospective controlled hip fracture study with 206 consecutive patients. Calcif. Tissue Int..

[5] Florence C.S., Bergen G., Atherly A., Burns E., Stevens J., Drake C. (2018). Medical costs of fatal and nonfatal falls in older adults. J. Am. Geriatr. Soc..

[6] Moreland B., Lee R. (2021). Emergency department visits and hospitalizations for selected nonfatal injuries among adults aged ≥65 years—United States, 2018. MMWR-Morb. Mortal. Wkly. Rep..

[7] Legters K. (2002). Fear of Falling. Phys. Ther..

[8] Burns E.R., Stevens J.A., Lee R. (2016). The direct costs of fatal and non-fatal falls among older adults—United States. J. Saf. Res..

[9] Heinrich S., Rapp K., Rissmann U., Becker C., König H.H. (2010). Cost of falls in old age: A systematic review. Osteoporos. Int..

[10] Vieira E.R., Palmer R.C., Chaves P.H.M. (2016). Prevention of falls in older people living in the community. BMJ.

[11] Wolinsky F.D., Johnson R.J., Fitzgerald J.F. (1992). Falling, health status, and the use of health services by older adults. A prospective study. Med. Care.

[12] Dunn J.E., Furner S.E., Miles T.P. (1993). Do falls predict institutionalization in older persons? An analysis of data from the longitudinal study of aging. J. Aging Health.

[13] Nunan S., Brown Wilson C., Henwood T., Parker D. (2018). Fall risk assessment tools for use among older adults in long-term care settings: A systematic review of the literature. Australas. J. Ageing.

[14] Guimaraes R.M., Isaacs B. (1980). Characteristics of the gait in old people who fall. Int. Rehabil. Med..

[15] Vlaeyen E., Stas J., Leysens G., Van der Elst E., Janssens E., Dejaeger E., Dobbels F., Milisen K. (2017). Implementation of fall prevention in residential care facilities: A systematic review of barriers and facilitators. Int. J. Nurs. Stud..

[16] Hausdorff J.M. (2005). Gait variability: Methods, modeling and meaning. J. Neuroeng. Rehabil..

[17] Park Y., Bae Y. (2025). Association Between Physical Performance, Gait Variability, and Fall Risk in Community-Dwelling Older Adults: Predictive Validity of Step-Width Variability for Screening of Fall Risk. Life.

[18] Kim U., Lim J., Park Y., Bae Y. (2025). Predicting fall risk through step width variability at increased gait speed in community-dwelling older adults. Sci. Rep..

[19] Yoshiko A., Hirono T., Takeda R., Chosa N., Beppu M., Watanabe K. (2023). Applicability of the seated step test for assessing thigh muscle sarcopenia in older individuals. Exp. Gerontol..

[20] Shin S., Demura S. (2009). Relationship between the Step Test with Stipulated Tempos and Gait Ability in the Elderly. J. Physiol. Anthropol..

[21] Kegelmeyer D., Minarsch R., Marita K., Hoffmeister A., Schnaterbeck G., Wohl T., Gokun Y., Kloos A. (2024). Step Test Evaluation of Performance on Stairs (STEPS): Assessing Stair Negotiation Ability in Older Adults. J. Geriatr. Phys. Ther..

[22] USAging (2022). Caregiver Needed: How the Nation’s Workforce Shortages Make It Harder to Age Well at Home.

[23] Chapman S.A., Greiman L., Bates T., Wagner L.M., Lissau A., Toivanen-Atilla K., Sage R. (2022). Personal Care Aides: Assessing self-care needs and worker shortages in rural areas. Health Aff..

[24] Brookings Institution *Immigration to Address the Caregiving Shortfall*; Brookings: Washington, DC, USA, 2024. https://www.brookings.edu/research/immigration-to-address-the-caregiving-shortfall.

[25] Wada N., Tsuchida W., Matanoki N., Mine Y., Kobayashi Y. (2025). Development of a fall risk assessment method for older adults using variability in stepping rhythm while seated: Focusing on safe movements in daily life. Biomechanisms.

[26] Hausdorff J.M., Rios D.A., Edelberg H.K. (2001). Gait variability and fall risk in community-living older adults: A 1-year prospective study. Arch. Phys. Med. Rehabil..

[27] Dubbeldam R., Lee Y.Y., Pennone J., Mochizuki L., Le Mouel C. (2023). Systematic review of candidate prognostic factors for Falling in older adults identified from Motion Analysis of challenging walking tasks. Eur. Rev. Aging Phys. Act..

[28] Terrier P., Reynard F. (2015). Effect of age on the variability and stability of gait: A cross-sectional treadmill study in healthy individuals between 20 and 69 years of age. Gait Posture.

[29] Ministry of Health, Labour and Welfare (Japan) *Long-Term Care Insurance System of Japan*; Ministry of Health, Labour and Welfare: Tokyo, Japan, 2021. https://www.mhlw.go.jp/english/policy/care-welfare/care-welfare-elderly/index.html.

[30] (1987). Kellogg International Work Group on the Prevention of Falls by the Elderly. The prevention of falls in later life: A report of the Kellogg international work group on the prevention of falls by the elderly. Dan. Med. Bull..

[31] Olsson-Collentine A., van Assen M.A.L.M., Hartgerink C.H.J. (2019). The prevalence of marginally significant results in psychology over time. Psychol. Sci..

[32] Cohen J. (1988). Statistical Power Analysis for the Behavioral Science.

[33] Tinetti M.E., Speechley M., Ginter S.F. (1988). Risk factors for falls among elderly persons living in the community. N. Engl. J. Med..

[34] Modarresi S., Divine A., Grahn J.A., Overend T.J., Hunter S.W. (2019). Gait parameters and characteristics associated with increased risk of falls in people with dementia: A systematic review. Int. Psychogeriatr..

[35] Blin O., Ferrandez A.M., Serratrice G. (1990). Quantitative analysis of gait in Parkinson patients: Increased variability of stride length. J. Neurol. Sci..

[36] Hausdorff J.M., Cudkowicz M.E., Firtion R., Wei J.Y., Goldberger A.L. (1998). Gait variability and basal ganglia disorders: Stride-to-stride variations of gait cycle timing in Parkinson’s disease and Huntington’s disease. Mov. Disord..

[37] Yamada M. (2012). Tailor-made fall prevention for the elderly. Res. Exerc. Epidemiol..

[38] Coughlin S.S. (1990). Recall bias in epidemiologic studies. J. Clin. Epidemiol..

